# Delta-Like Ligand 3 Expression and Functional Imaging in Gastroenteropancreatic Neuroendocrine Neoplasms

**DOI:** 10.1200/PO-25-00724

**Published:** 2025-11-20

**Authors:** Rohit Thummalapalli, Salomon Tendler, Joanne F. Chou, Zeynep C. Tarcan, Courtney Porfido, Jonathan Willner, Irina Linkov, Umesh Bhanot, Alissa J. Cooper, Jierui Xu, James J. Harding, Natasha Rekhtman, Laura H. Tang, Charles M. Rudin, Yelena Y. Janjigian, Heiko Schöder, John T. Porier, Jinru Shia, Olca Basturk, Diane Reidy-Lagunes, Marinela Capanu, Jason S. Lewis, Lisa Bodei, Mark P. Dunphy, Nitya Raj

**Affiliations:** ^1^ Department of Medicine, Memorial Sloan Kettering Cancer Center, New York, NY; ^2^ Department of Epidemiology and Biostatistics, Memorial Sloan Kettering Cancer Center, New York, NY; ^3^ Department of Pathology and Laboratory Medicine, Memorial Sloan Kettering Cancer Center, New York, NY; ^4^ Pathology Core Facility, Department of Pathology and Laboratory Medicine, Memorial Sloan Kettering Cancer Center, New York, NY; ^5^ Marie-Josée and Henry R. Kravis Center for Molecular Oncology, Memorial Sloan Kettering Cancer Center, New York, NY; ^6^ Department of Radiology, Memorial Sloan Kettering Cancer Center, New York, NY; ^7^ Perlmutter Cancer Center, New York University Langone Health, New York, NY

## Abstract

**PURPOSE:**

Delta-like ligand 3 (DLL3) is an emerging target across neuroendocrine cancers, but remains underexplored in gastroenteropancreatic neuroendocrine neoplasms (GEP NENs), including poorly differentiated gastroenteropancreatic neuroendocrine carcinomas (GEP NECs) and well-differentiated neuroendocrine tumors (NETs). We aimed to define the landscape of DLL3 expression and feasibility of DLL3-targeted imaging in this population.

**PATIENTS AND METHODS:**

We completed DLL3 immunohistochemistry (IHC) on 379 tumor samples from patients with GEP NENs, analyzing associations between DLL3 IHC positivity, clinicopathologic features, and outcomes. [^89^Zr]Zr-DFO-SC16.56 DLL3 immuno-positron emission tomography-computed tomography (immunoPET-CT) imaging was performed in six patients with DLL3 IHC-positive advanced GEP NENs.

**RESULTS:**

Among GEP NECs, DLL3 expression was identified in 55/78 (71%) tumors, was enriched for small cell histology, and did not demonstrate prognostic significance. Among well-differentiated gastroenteropancreatic neuroendocrine tumors, DLL3 expression was identified in 5/235 (2%) of grade 1-2 and 25/66 (40%) grade 3 (G3) tumors, most commonly G3 pancreatic NETs (PanNETs; 22/52, 43%), with univariate analysis revealing increased mortality risk among patients with DLL3-positive advanced G3 PanNETs (hazard ratio 3.27 [95% CI, 1.09 to 9.78]). Between May 28, 2024, and February 10, 2025, six patients with DLL3 IHC-positive GEP NENs underwent [^89^Zr]Zr-DFO-SC16.56 immunoPET-CT imaging, which delineated DLL3-avid tumor lesions in five of six patients (two of two GEP NECs, three of four G3 PanNETs). Tumor-specific uptake of [^89^Zr]Zr-DFO-SC16.56 varied between patients, with maximum standard uptake values ranging from 7.4 to 36.7, with four of six cases demonstrating DLL3 avidity in ≥50% of tumor lesions.

**CONCLUSION:**

DLL3 is expressed on a majority of GEP NECs and on a subset of high-grade PanNETs marked by poor outcomes. Functional imaging suggests DLL3 as a promising therapeutic target in both GEP NECs and high-grade PanNETs.

## INTRODUCTION

Delta-like ligand 3 (DLL3) is an inhibitory Notch pathway ligand and a target of achaete-scute homolog 1,^1^ a critical transcription factor driving neuroendocrine cell fate decisions in small cell lung cancer (SCLC).^2^ DLL3 exhibits high cell surface expression in SCLC and neuroendocrine prostate cancer (NEPC),^3,4^ which has prompted efforts to target DLL3^5^ including DLL3-targeted T-cell engagers (TCEs),^6-8^ antibody-drug conjugates,^9^ and radioligand therapies.^10,11^ Notably, tarlatamab, a DLL3-CD3 bispecific TCE, recently demonstrated benefit over chemotherapy in patients with previously treated SCLC.^12^ Moreover, recent descriptions of functional DLL3 radiotracer uptake in patients with SCLC and NEPC^13^ have prompted exploration of DLL3 radioligand therapies.^10,11^

**CONTEXT**

**Key Objective**
To evaluate the landscape of delta-like ligand 3 (DLL3) expression in gastroenteropancreatic neuroendocrine neoplasms (GEP NENs) and define opportunities for DLL3 therapeutic targeting in these populations.
**Knowledge Generated**
In this cohort of 379 patients with GEP NENs, DLL3 immunohistochemistry (IHC) expression was detected in a majority of poorly differentiated gastroenteropancreatic neuroendocrine carcinomas (GEP NECs) and a subset of grade 3 (G3) pancreatic neuroendocrine tumors (PanNETs) associated with poor clinical outcomes. DLL3 immuno-positron emission tomography (PET)-computed tomography imaging revealed high level DLL3 avidity in patients with GEP NECs and subsets of patients with G3 PanNETs, supporting rationale for therapeutic targeting.
**Relevance**
IHC and PET imaging revealed DLL3 expression in a majority of GEP NECs and in an aggressive subset of high-grade PanNETs. Future DLL3 therapeutic efforts focused on the GEP NEC and high-grade NET populations are warranted.

Despite advances in SCLC and NEPC, the role for DLL3-targeted therapies in patients with other extrapulmonary neuroendocrine neoplasms remains underexplored. Gastroenteropancreatic neuroendocrine neoplasms (GEP NENs), which include well-differentiated gastroenteropancreatic neuroendocrine tumors (GEP NETs) and poorly differentiated gastroenteropancreatic neuroendocrine carcinomas (GEP NECs), are now the second most common GI malignancies^14^ and are in need of new therapeutic options. Patients with GEP NETs are often treated with somatostatin receptor (SSTR)–targeted therapies, including somatostatin analogs^15,16^ and SSTR-targeted radioligand therapies (^177^Lu-DOTATATE^17,18^), targeted therapies,^19,20^ and chemotherapies.^21,22^ However, the optimal management of high-grade (grade 3 [G3]) GEP NETs remains unclear. These tumors often show lower and/or more heterogeneous SSTR positron emission tomography (PET) avidity compared with grade 1-2 (G1-G2) NETs and are often treated with chemotherapies; however, response rates to chemotherapies are generally lower in G3 GEP NETs compared with GEP NECs.^23^ By contrast, GEP NECs are uniformly aggressive malignancies generally treated with platinum-based chemotherapies. In sum, new therapies are needed for both G3 GEP NETs and GEP NECs.

Given the success of SSTR-targeted radioligands in patients with GEP NENs,^17,18^ whether DLL3- or other cell surface antigen–directed therapies can be similarly effective is of key interest, particularly in the G3 GEP NET and GEP NEC populations, in whom benefits of SSTR-targeted radioligand therapies are often limited.^24,25^ Furthermore, the degree of DLL3 expression required for actionability of DLL3-targeted therapies and the degree of DLL3 intertumoral heterogeneity in patients with GEP NENs remain unknown, for which development of a functional, dynamic, patient-level marker of DLL3 expression is required. Here, we describe the landscape of DLL3 immunohistochemistry (IHC) expression in patients with GEP NENs and feasibility of [^89^Zr]Zr-DFO-SC16.56 functional DLL3 PET imaging,^13^ to further define opportunities for DLL3 therapeutic targeting in these populations.

## PATIENTS AND METHODS

### Patients, Tissue Samples, and Clinicopathologic Analyses

All patients with histologically confirmed poorly differentiated GEP NECs and G3 well-differentiated GEP NETs treated between January 1, 2018, and July 1, 2025, at Memorial Sloan Kettering (MSK) Cancer Center and with available pathology specimens were identified, with the results of DLL3 IHC performed prospectively as below collected. Patients with GEP NECs and G3 GEP NETs in whom DLL3 IHC was not performed prospectively were profiled retrospectively using formalin-fixed, paraffin-embedded tissue slides or tissue microarrays (TMAs; two 1.5-mm cores per sample from a representative tumor area). DLL3 IHC was also performed on TMAs constructed from resected G1-G2 small bowel, pancreatic, esophagogastric, and colorectal NETs treated between 2018 and 2024, G1-G2 pancreatic NETs (PanNETs) treated between 2002 and 2009 (Willner et al manuscript submitted for publication), and G1 small bowel NETs treated between 1995 and 2008 (Willner et al manuscript submitted for publication). For patients with available DLL3 IHC data, clinicopathologic information, Memorial Sloan Kettering-Integrated Molecular Profiling of Actionable Cancer Targets^26^ next-generation sequencing data, and systemic therapy outcomes were collected. The data cutoff for retrospective analyses was July 1, 2025. This portion of the study was approved by the Institutional Review Board/Privacy Board at MSK and followed the Belmont report for retrospective review of records and waiver of consent.

### DLL3 IHC

Tumor samples were assessed for DLL3 expression by IHC using a standardized Ventana platform assay (clone SP347, Ventana, Roche, Tucson, AZ).^27^ DLL3 expression was quantified on the basis of estimation of the percentage of positive tumor cells (range, 0%-100%) multiplied by staining intensity (weak: 1+, moderate: 2+, strong: 3+) to generate an H-score (range, 0-300). Positive DLL3 expression was defined as ≥5% of tumor cells with 1+ or higher staining. All staining assessments were completed by board-certified pathologists (J.S., O.B., L.H.T., Z.C.T., and U.B.).

### Retrospective Evaluation of ^68^Ga-DOTATATE PET-Computed Tomography (CT) Characteristics

Among patients with PanNETs who underwent ^68^Ga-DOTATATE PET-CT imaging within 6 months of tumor tissue collection for DLL3 IHC, a board-certified radiologist (L.B.) evaluated ^68^Ga-DOTATATE PET-CT characteristics of tumor lesions at corresponding sites of tumor tissue evaluation. For each patient, the maximum standard uptake value (SUV_max_), and tumor/liver and tumor/spleen ratios for the corresponding tumor lesion were identified by evaluating SUV_mean_ for liver and spleen background, when available. Patients with intercurrent local therapies or systemic therapy changes between tumor tissue collection for DLL3 IHC and ^68^Ga-DOTATATE PET-CT were excluded.

### [^89^Zr]Zr-DFO-SC16.56 DLL3 immunoPET-CT Imaging

Between May 28, 2024, and February 10, 2025, selected patients with advanced DLL3 IHC-positive GEP NENs with progression of disease within ≤12 weeks on systemic therapy were enrolled onto a phase II study of the humanized anti-DLL3 monoclonal antibody SC16.56 labeled with Zirconium-89 (^89^Zr) for PET imaging (ClinicalTrials.gov identifier: NCT04199741), as previously described.^13^ Patients received an intravenous injection of [^89^Zr]Zr-DFO-SC16.56, which included 2.5 mg of SC16.56 and 37-74 MBq of ^89^Zr, followed by a PET-CT 3-5 days later.^13^ Images were analyzed by board-certified nuclear medicine physicians (M.P.D. and L.B.) as previously described.^13^ Tumoral uptake was reported as SUV_max_ and SUV_mean_. For quantitative tumor analyses, the lesion with the highest SUV was selected from each patient, with tumors with tracer retention greater than blood pool considered avid. In addition, the number and percentage of DLL3-positive tumor lesions were calculated comparing [^89^Zr]Zr-DFO-SC16.56 DLL3 PET imaging with results of recent standard-of-care scans including CT or ^68^Ga-DOTATATE PET-CT within ≤12 weeks, with inclusion criterion for evaluable tumor lesions including soft tissue lesions >1.5 cm. All patients provided written informed consent per the Declaration of Helsinki.

### Statistical Analyses

Covariate distributions between DLL3-positive and DLL3-negative groups were compared using Fisher exact tests (categorical variables) and Wilcoxon rank-sum tests (continuous variables). For genomic enrichment analyses, Fisher exact tests were used to calculate *P* values for differences in frequency of OncoKB^28^ oncogenic alterations between DLL3 groups; *Q* values were calculated using the false discovery rate/Benjamini-Hochberg method and corrected for the number of genes with ≥5% oncogenic alterations in each cohort. For clinical outcome analyses, overall survival (OS) was calculated from the date of tissue collection, where DLL3 IHC status was ascertained, until the date of last follow-up or death. Among patients with advanced GEP NECs, progression-free survival (PFS) with first-line platinum-based chemotherapy was calculated from the date of starting therapy until date of first disease progression or death. PFS and OS were estimated using Kaplan-Meier methods and compared between groups using log-rank tests. Among advanced G3 PanNETs and advanced PanNETs (all grades), univariate Cox regression models were used to correlate DLL3 IHC status with OS from the date of diagnosis of advanced G3 disease or advanced disease of any grade, respectively, incorporating DLL3 IHC status as a time-dependent covariate. All analyses were performed using R Version 4.3.2. All *P* values were two-sided, with <0.05 considered statistically significant.

## RESULTS

### Landscape of DLL3 Expression in GEP NECs

**FIG 1.**
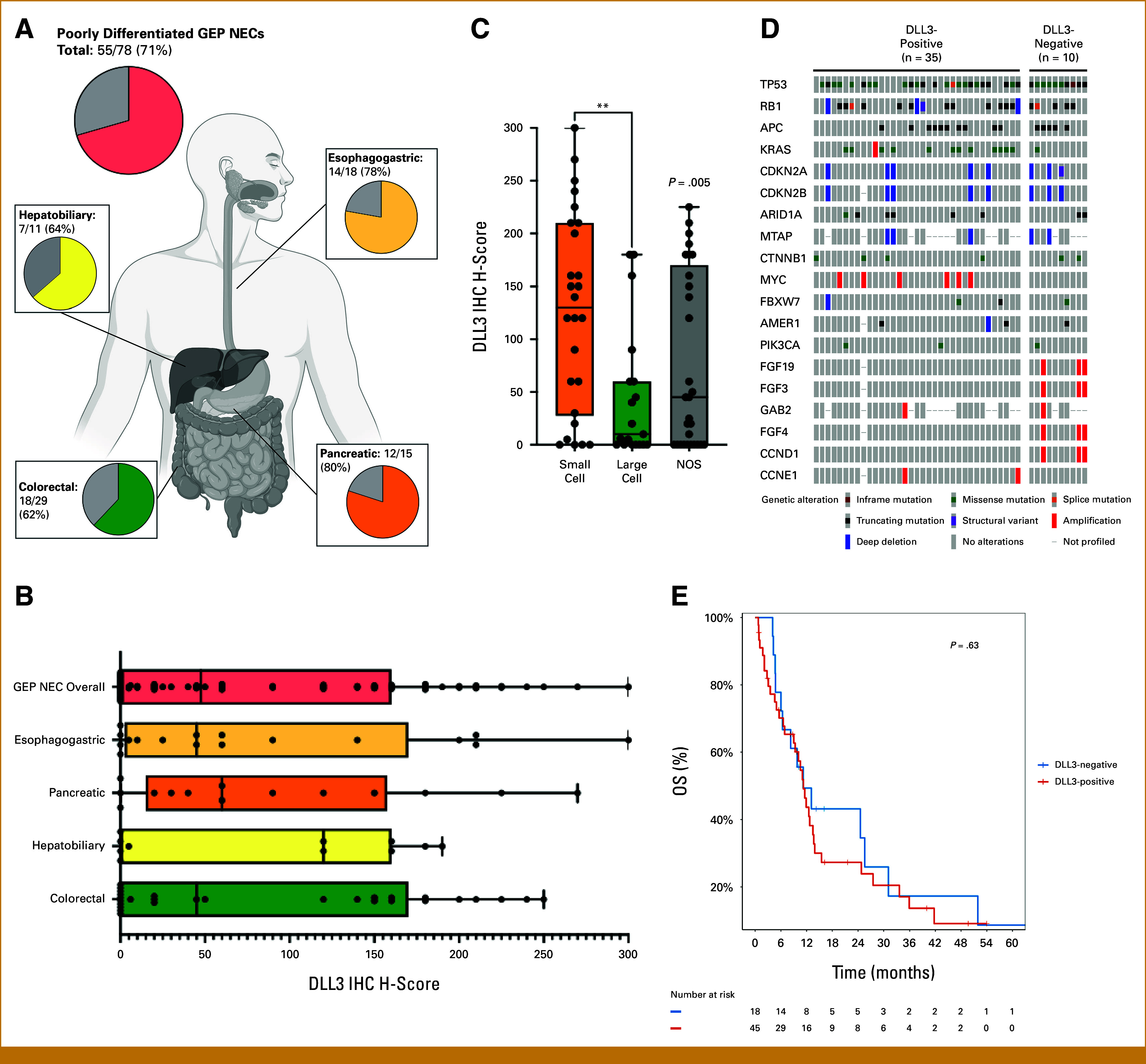
Landscape of DLL3 IHC expression and clinicopathologic correlates of DLL3 expression in poorly differentiated GEP NECs. (A) DLL3 IHC positivity rates among GEP NECs overall and by primary site of disease. (B) Box and whisker plots describing median, IQR, and distribution of individual DLL3 IHC H-scores among tumor samples from poorly differentiated GEP NECs overall and by primary site of origin. (C) Box and whisker plots describing median, IQR, and distribution of individual DLL3 IHC H-scores among GEP NEC tumor samples with small cell histology (n = 26), large cell histology (n = 21), or histology NOS (n = 31). *P* value refers to comparison between small cell and large cell groups by Wilcoxon rank-sum test. (D) OncoPrint of genomic alterations by DLL3 status among GEP NEC tumors with available DLL3 IHC and MSK-IMPACT NGS. Only genes with at least 5% prevalence of oncogenic or likely oncogenic alterations in the cohort are displayed. (E) OS from diagnosis of advanced disease by DLL3 IHC status among patients with advanced GEP NECs. *P* value corresponds to log-rank test. DLL3, delta-like ligand 3; GEP NEC, gastroenteropancreatic neuroendocrine carcinoma; IHC, immunohistochemistry; MSK-IMPACT, Memorial Sloan Kettering-Integrated Molecular Profiling of Actionable Cancer Targets; NGS, next-generation sequencing; NOS, not otherwise specified; OS, overall survival.

DLL3 expression was detected in 55 of 78 GEP NEC samples overall (71%; median IHC H-score, 47.5; IQR, 0-160; range, 0-300); breakdowns by GEP NEC subtype and by DLL3-positive cases only are displayed in Figures 1A and 1B and Table 1. Median H-score was significantly higher in small cell compared with large cell histology (130 *v* 10, *P* = .005; Fig 1C and Data Supplement, Table S1), and median Ki-67 proliferative index was higher among DLL3-positive compared with DLL3-negative samples (90 *v* 75, *P* = .044; Data Supplement Fig S1A and Table S1). No significant genomic differences were observed between DLL3-positive and DLL3-negative tumors overall (Fig 1D) or within any GEP NEC subtype (Data Supplement, Table S2).

**TABLE 1.** DLL3 IHC Expression in GEP NENs

Tumor Type	DLL3-Positive (%)	DLL3 IHC H-Score (median, IQR): All Tumors	DLL3 IHC H-Score (median, IQR): DLL3-Positive Tumors Only
GEP NEC overall	55/78 (71)	47.5 (0-160)	120 (45-180)
Esophagogastric	14/18 (78)	45 (2.5-170)	60 (35-205)
Pancreatic	12/15 (80)	60 (15-157.5)	90 (40-180)
Hepatobiliary	7/11 (64)	120 (0-160)	160 (120-180)
Colorectal	18/29 (62)	45 (0-170)	155 (48.8-202.5)
Other	4/5 (80)	20 (5-180)	40 (12.5-150)
PanNET			
G1 PanNET	3/46 (7)	0 (0-0)	8 (5-15)
G2 PanNET	1/23 (4)	0 (0-0)	180 (180-180)
G3 PanNET	22/52 (42)	0 (0-27.5)	45 (15-142.5)
SB NET			
G1 SB NET	0/137 (0)	0 (0-0)	NA
G2 SB NET	1/21 (5)	0 (0-0)	20 (20-20)
G3 SB NET	1/5 (20)	0 (0-10)	20 (20-20)
Esophagogastric NET			
G2 gastric NET	0/1 (0)	0 (0-0)	NA
G3 gastric NET	0/4 (0)	0 (0-0)	NA
Colorectal NET			
G1 colorectal NET	0/5 (0)	0 (0-0)	NA
G2 colorectal NET	0/2 (0)	0 (0-0)	NA
G3 colorectal NET	2/5 (40)	0 (0-39)	39 (8-70)

NOTE. Displayed are DLL3 IHC positivity rates among poorly differentiated GEP NECs overall and by primary site of disease, and well-differentiated PanNETs, SB NETs, esophagogastric, and colorectal NETs by grade (G1, G2, and G3). Other GEP NEC included SB NEC (n = 3) and NEC of unknown primary (n = 2). IHC positivity was defined as ≥5% of tumor cells with ≥weak (1+) IHC staining.

Abbreviations: DLL3, delta-like ligand 3; G1, grade 1; G2, grade 2; G3, grade 3; GEP NEC, gastroenteropancreatic neuroendocrine carcinoma; GEP NEN, gastroenteropancreatic neuroendocrine neoplasm; IHC, immunohistochemistry; NA, not applicable; NET, neuroendocrine tumor; PanNET, pancreatic NET; SB NEC, small bowel NEC; SB NET, small bowel NET.

We next sought to determine the prognostic significance of DLL3 expression in patients with advanced GEP NECs (n = 64; Data Supplement, Table S3). Among patients treated with first-line platinum-based chemotherapy (n = 37), PFS was not significantly different between patients with DLL3-positive and DLL3-negative tumors (median 2.9 *v* 4.3 months, *P* = .57; Data Supplement, Fig S3). Similarly, OS was not significantly different between patients with DLL3-positive and DLL3-negative tumors (median 11 *v* 11 months, *P* = .63; Fig 1E).

### Landscape of DLL3 Expression in Well-Differentiated GEP NETs

**FIG 2.**
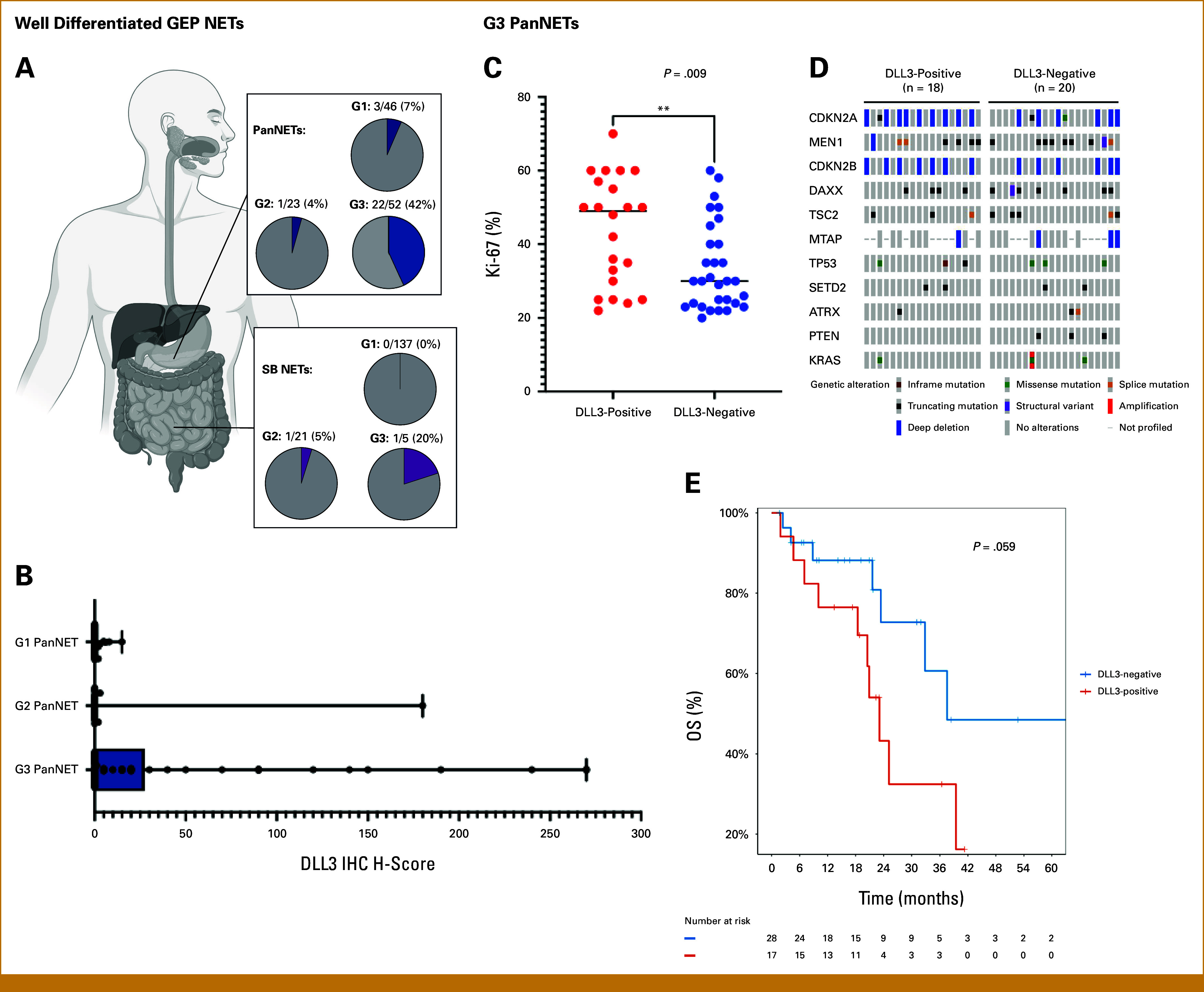
Landscape of DLL3 IHC expression in well-differentiated GEP NETs and clinicopathologic correlates of DLL3 expression in G3 well-differentiated PanNETs. (A) DLL3 IHC positivity rates among well-differentiated PanNETs and small bowel NETs by grade (G1, G2, G3). (B) Box and whisker plots describing median, IQR, and distribution of individual DLL3 IHC H-scores among tumor samples from PanNETs by grade. (C) Distribution of Ki-67 percentages (%) among G3 PanNETs by DLL3 (IHC) positive (n = 22) and negative (n = 30) status. *P* value refers to comparison between groups by Wilcoxon rank-sum test. (D) OncoPrint of genomic alterations by DLL3 status among G3 PanNET tumors with available DLL3 IHC and MSK-IMPACT NGS. Only genes with at least 5% prevalence of oncogenic or likely oncogenic alterations in the cohort are displayed. (E) OS from time of tumor evaluation by DLL3 IHC in patients with advanced G3 PanNETs. *P* value corresponds to log-rank test. DLL3, delta-like ligand 3; G1, grade 1; G2, grade 2; G3, grade 3; GEP NET, gastroenteropancreatic neuroendocrine tumor; IHC, immunohistochemistry; MSK-IMPACT, Memorial Sloan Kettering-Integrated Molecular Profiling of Actionable Cancer Targets; NGS, next-generation sequencing; OS, overall survival; PanNET, pancreatic NET.

Among 301 GEP NETs tested, DLL3 expression was largely confined to G3 tumors, with 5 of 235 (2%) G1-G2 tumors and 25 of 66 (38%) G3 tumors testing positive. These included 22 of 52 (43%) G3 PanNETs (median IHC H-score, 0; IQR, 0-27.5; range, 0-270), 1 of 5 (20%) G3 small bowel NETs, 0 of 4 (0%) G3 gastric NETs, and 2 of 5 (40%) G3 colorectal NETs (Figs 2A and 2B; Table 1).

Given that G3 PanNETs are more commonly observed in clinical practice,^29^ we next aimed to specifically define the landscape of DLL3 expression in PanNETs. Median Ki-67 was significantly higher in DLL3-positive tumors among PanNETs overall (45% *v* 8%, *P* < .001; Data Supplement, Fig S1B) and within G3 PanNETs specifically (49% *v* 30%, *P* = .009; Fig 2C). No significant genomic differences were observed between DLL3-positive and DLL3-negative G3 PanNETs (Fig 2D and Data Supplement, Table S4). Next, given the clear association of DLL3 positivity with high-grade disease (Figs 1A and 1C, Data Supplement, Fig S1B), we aimed to explore the correlation of DLL3 tumor IHC positivity with lesion-level SSTR PET avidity. Interestingly, among 24 patients with PanNETs who underwent concurrent ^68^Ga-DOTATATE PET-CT imaging, there was no association between tumor DLL3 IHC positivity and ^68^Ga-DOTATATE PET-CT characteristics from the corresponding tumor lesion (Data Supplement, Figs S4A and S4C), suggesting DLL3 expression in PanNETs is not mutually exclusive with SSTR PET avidity.

### Prognostic Significance of DLL3 Expression in PanNETs

Given association of DLL3 positivity with high-grade disease, we aimed to understand its prognostic significance among patients with advanced PanNETs (n = 66; Data Supplement, Table S5). We observed shorter OS in patients diagnosed with DLL3-positive advanced G3 PanNETs (*P* = .059; Fig 2E) as well as in DLL3-positive advanced PanNETs of all grades (*P* < .001; Data Supplement, Fig S5A), with systemic therapy regimens generally balanced between groups (Data Supplement, Table S5). In addition, using a univariate Cox regression model incorporating DLL3 IHC status as a time-dependent covariate, we observed significantly increased risk for mortality from the time of diagnosis of G3 advanced disease among DLL3-positive patients (OS hazard ratio [HR] 3.27 [95% CI, 1.09 to 9.78]; *P* = .034). Similar results were observed when considering advanced PanNETs of all grades (OS HR 4.53 [95% CI, 1.86 to 11.0]; *P* < .001).

### DLL3 immunoPET-CT Imaging Reveals DLL3 as an Actionable Target in GEP NENs

**FIG 3.**
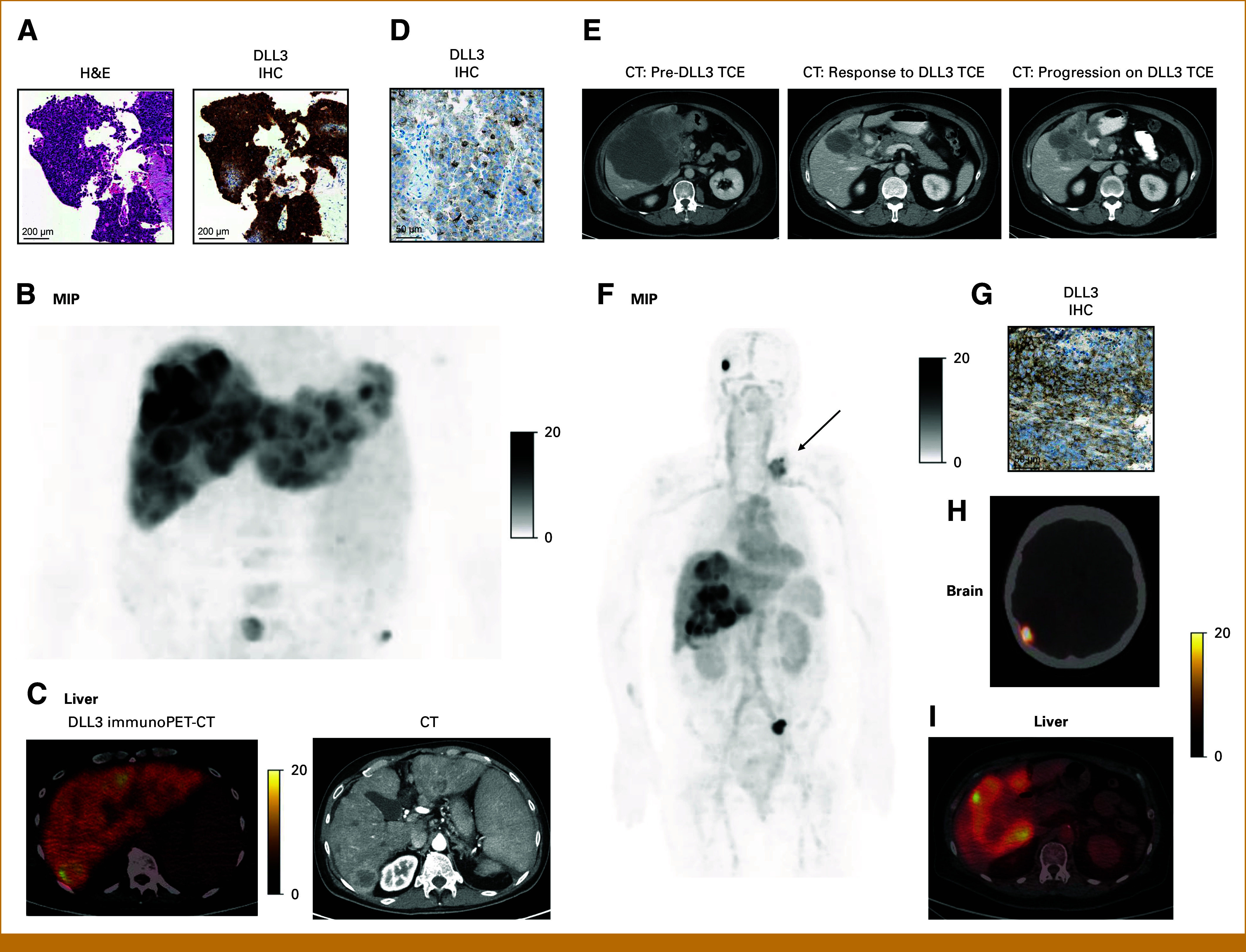
[^89^Zr]Zr-DFO-SC16.56 DLL3 immunoPET-CT imaging in patients with advanced poorly differentiated GEP NECs. (A-C) A patient with poorly differentiated pancreatic NEC metastatic to liver with previous progression of disease on carboplatin and etoposide and CAPOX underwent [^89^Zr]Zr-DFO-SC16.56 DLL3 immunoPET-CT imaging (patient 1). (A) H&E and DLL3 IHC images from a liver metastasis biopsy before start of systemic therapy, revealing DLL3 IHC H-score of 270. (B) MIP and (C) axial images from DLL3 immunoPET-CT, with corresponding CT completed 2 days after DLL3 immunoPET-CT. (D-I) A patient with gallbladder NEC with multiple previous lines of systemic therapy underwent [^89^Zr]Zr-DFO-SC16.56 DLL3 immunoPET-CT imaging after previous progression of disease on DLL3 TCE therapy (patient 2). (D) DLL3 IHC from a liver metastasis at biopsy confirmation of NEC histology before DLL3 TCE therapy revealed IHC H-score of 160. (E) Serial CT scans on DLL3 TCE therapy revealing partial response followed by progression of disease. At progression of disease on fourth-line carboplatin/etoposide, [^89^Zr]Zr-DFO-SC16.56 DLL3 immunoPET-CT was completed, with (F) MIP and (H and I) axial images highlighting brain and liver metastases. (G) Biopsy of DLL3 PET-avid left supraclavicular nodal metastasis (corresponding to arrow in MIP image) revealed retained DLL3 IHC expression (H-score 120). For MIP and axial images, SUV scales are displayed. For MIP images, pixels in PET imaging with SUV 0 appear white and pixels with SUV ≥ upper thresholds appear black. For axial images, pixels with SUV 0 and SUV ≥ upper thresholds appear black and bright yellow, respectively. CAPOX, capecitabine and oxaliplatin; CT, computed tomography; DLL3, delta-like ligand 3; GEP NEC, gastroenteropancreatic neuroendocrine carcinoma; H&E, hematoxylin and eosin; IHC, immunohistochemistry; MIP, maximum-intensity projection; PET, positron emission tomography; SUV, standard uptake value; TCE, T-cell engager.

**FIG 4.**
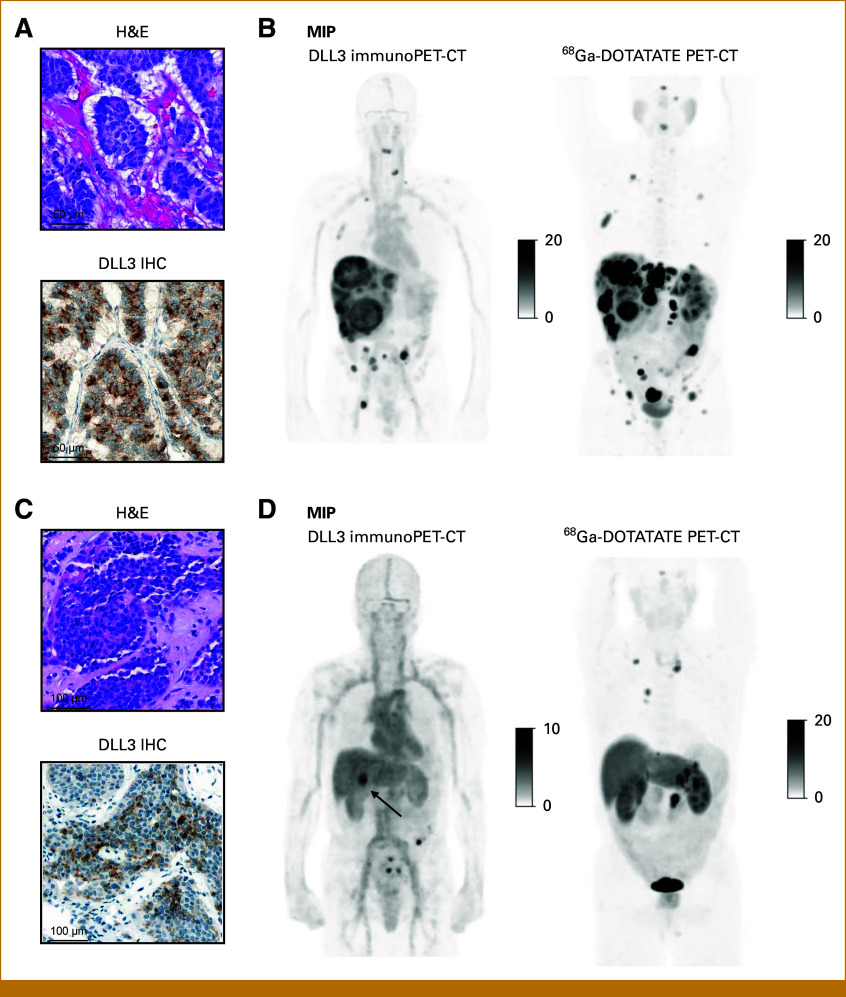
[^89^Zr]Zr-DFO-SC16.56 DLL3 immunoPET-CT imaging in patients with advanced G3 well-differentiated PanNETs. (A and B) A patient with G3 PanNET metastatic to liver underwent [^89^Zr]Zr-DFO-SC16.56 DLL3 immunoPET-CT imaging at progression of disease on third-line ^177^Lu-DOTATATE (patient 5). (A) H&E and DLL3 IHC images from the patient's pancreatic primary tumor before start of systemic therapy. (B) MIP images from DLL3 immunoPET-CT and ^68^Ga-DOTATATE PET-CT imaging completed 12 weeks before DLL3 immunoPET-CT, revealing 100% of tumor lesions positive for both DLL3 and SSTR avidity. (C and D) A patient with G3 PanNET metastatic to liver, lung, and mediastinum underwent DLL3 immunoPET-CT after progression of disease in the liver on second-line capecitabine and temozolomide. (C) H&E and DLL3 IHC images from progressive liver metastases, revealing DLL3 IHC 120. (D) MIP images from DLL3 immunoPET-CT and ^68^Ga-DOTATATE PET-CT imaging completed 5 weeks before DLL3 immunoPET-CT, revealing DLL3 uptake in the progressive liver metastasis (SUV_max_ 14.4); the remainder of the disease was otherwise DLL3 PET-negative and SSTR PET-positive. The arrow notes progressive liver metastatic lesion, which was biopsied for DLL3 IHC evaluation. For all images, SUV scales are displayed, with pixels in PET imaging with SUV 0 appearing white and pixels with SUV ≥ upper thresholds appearing black. CT, computed tomography; DLL3, delta-like ligand 3; G3, grade 3; H&E, hematoxylin and eosin; IHC, immunohistochemistry; MIP, maximum-intensity projection; PanNET, pancreatic neuroendocrine tumor; PET, positron emission tomography; SSTR, somatostatin receptor; SUV, standard uptake value.

[^89^Zr]Zr-DFO-SC16.56 immunoPET-CT imaging^13^ was performed in six patients with advanced DLL3 IHC-positive GEP NENs (Table 2 and Data Supplement, Table S6). In total, tumor-specific [^89^Zr]Zr-DFO-SC16.56 uptake was observed in five of six patients, including both patients with GEP NECs and three of four patients with G3 PanNETs, with minimal background uptake (Table 2, Figs 3 and 4, and Data Supplement, Fig S6). No adverse events were observed (Data Supplement, Table S6).

**TABLE 2.** Patient Characteristics and Results of [^89^Zr]Zr-DFO-SC16.56 DLL3 immunoPET-CT Imaging

Patient No.	Tumor Type	DLL3 IHC H-Score (biopsy location)	No. of Previous Lines of Systemic Therapy for Advanced Disease	Most Recent Systemic Therapy Before DLL3 ImmunoPET CT	DLL3 ImmunoPET-CT–Avid	SUV_max_ of Highest DLL3-Avid Lesion (location)	Blood Pool Uptake, SUV_mean_	Tumor/Liver Ratio, SUV_max_/SUV_mean_	No. (%) of Tumor Lesions DLL3 ImmunoPET-CT–Positive	Among DLL3 immunoPET-CT–Positive Tumor Lesions, ^68^Ga-DOTATATE–Positive (PanNET only, %)
1	Pancreatic NEC	270 (liver)	2	CAPOX	Yes	36.7 (liver)	1.6	5.7	20/21 (95)	NA
2	Gallbladder NEC	120 (supraclavicular lymph node)	4	Carboplatin + etoposide	Yes	32.4 (liver)	7.9	4.8	16/16 (100)	NA
3	G3 PanNET	90 (liver)	4	Capecitabine + temozolomide	No	7.4 (abdominal lymph node)	7.9	1.1	0/22 (0)	NA
4	G3 PanNET	120 (liver)	2	Capecitabine + temozolomide	Yes	14.4 (liver)	4.8	3.1	1/10 (10)	0
5	G3 PanNET	90 (pancreatic primary)	3	^177^Lu-DOTATATE	Yes	27.5 (liver)	3.9	5.2	38/38 (100)	100
6	G3 PanNET	30 (liver)	3	CAPOX	Yes	15.0 (liver)	5.5	2.7	4/8 (50)	100

Abbreviations: CAPOX, capecitabine and oxaliplatin; CT, computed tomography; DLL3, delta-like ligand 3; G3, grade 3; IHC, immunohistochemistry; NA, not applicable; NEC, neuroendocrine carcinoma; PanNET, pancreatic neuroendocrine tumor; PET, positron emission tomography; SUV, standard uptake value.

### DLL3 immunoPET-CT Imaging in Patients With GEP NECs

Two patients with GEP NECs were imaged. Patient 1 (Figs 3A and 3C) was a patient with advanced pancreatic small cell NEC who experienced recent progression on second-line chemotherapy with DLL3 IHC from a progressing tumor lesion demonstrating H-score of 270 (Fig 3A). High-level tumor-specific [^89^Zr]Zr-DFO-SC16.56 uptake was demonstrated, with DLL3 PET SUV_max_ of 36.7, and 95% of tumor lesions demonstrating DLL3 PET avidity (Figs 3B and 3C). Patient 2 (Figs 3C and 3I) was initially diagnosed with a primary gallbladder carcinoma. At progression on second-line chemotherapy, repeat liver lesion biopsy revealed high-grade NEC, with DLL3 IHC H-score of 160 (Fig 3D). As a result, the patient was treated with a DLL3 bispecific TCE with clinical and radiographic response (Fig 3E). The patient ultimately developed progressive disease on DLL3 TCE therapy, prompting switch in therapy. At further progression, the patient underwent [^89^Zr]Zr-DFO-SC16.56 DLL3 PET imaging, which revealed avidity in all active tumor lesions, with highest uptake observed in a progressing liver metastasis (SUV_max_, 32.4; Figs 3F, 3H, and 3I). A progressive left supraclavicular lymph node demonstrated SUV_max_ of 10.9, prompting biopsy revealing DLL3 IHC H-score 120 (Fig 3G). In addition, a DLL3-avid asymptomatic brain metastasis was identified (SUV_max_, 29.1; Fig 3H). Given confirmation of uniformly retained high-level DLL3 PET avidity after progression on prior DLL3 TCE therapy, the patient was considered for treatment with a DLL3-directed antibody-drug conjugate.

### DLL3 immunoPET-CT Imaging in Patients With High-Grade PanNETs

DLL3 immunoPET-CT imaging was completed in four patients with DLL3 IHC-positive advanced G3 PanNETs (IHC H-score range, 30-120), with degree of tracer avidity and interlesional heterogeneity more variable. Among these patients, three of four demonstrated [^89^Zr]Zr-DFO-SC16.56 uptake, ranging from SUV_max_ 14.4 to 27.5, with 10% to 100% of tumor lesions demonstrating DLL3 PET avidity (Table 2). These included a patient with G3 PanNET with recent progression of disease on ^177^Lu-DOTATATE and previous DLL3 IHC H-score 90, in whom DLL3 immunoPET-CT revealed 100% of active tumor lesions positive for DLL3 tracer uptake with SUV_max_ of 27.5 in a progressing liver lesion (patient 5; Table 2, Figs 4A and 4B). In two of three patients (patients 5 and 6; Figs 4A and 4B, Data Supplement, Fig S6B), DLL3 immunoPET-CT imaging revealed that all individual DLL3 PET-positive tumor lesions also demonstrated SSTR avidity on corresponding ^68^Ga-DOTATATE PET-CT imaging. Conversely, we observed one patient with advanced G3 PanNET and recent hepatic progression on capecitabine and temozolomide (patient 4; Figs 4C and 4D), with a previous liver metastasis biopsy revealing DLL3 IHC H-score 120 (Fig 4C). In this patient, the progressive liver lesion was positive on [^89^Zr]Zr-DFO-SC16.56 PET imaging (SUV_max_, 14.4) but was not visualized on concurrent SSTR imaging, whereas the remainder of the disease was DLL3 PET-negative and SSTR PET-positive (Fig 4D). Overall, these suggested DLL3 avidity can be observed both alongside and independently of SSTR avidity in patients with PanNETs.

## DISCUSSION

Here, using IHC and functional PET imaging, we describe the clinical landscape of DLL3 expression in GEP NENs. We demonstrate the potential for DLL3-directed therapy development in poorly differentiated GEP NECs and subsets of well differentiated GEP NETs, particularly high-grade PanNETs, and highlight potential utility of [^89^Zr]Zr-DFO-SC16.56 DLL3 immunoPET-CT imaging for guiding therapy selection.

Our demonstration of DLL3 IHC expression in a majority of GEP NECs adds to the growing body of literature describing the prevalence of DLL3 expression in these tumors. Similar recent efforts have estimated DLL3 expression ranging from 44% to 77% in GEP NECs^30-32^; notably, thresholds for DLL3 IHC positivity have varied across studies. In concert with recent studies,^31,32^ we observed association of DLL3 expression with small cell histology and identified no prognostic significance for DLL3 expression. Given high prevalence of DLL3 expression, our data provide support for ongoing DLL3 therapeutic trials in GEP NECs^33,34^ and provide rationale for further drug development in patients with these tumors.

In contrast to GEP NECs, previous studies have reported low DLL3 expression in well-differentiated GEP NETs,^30,35^ suggesting minimal role for DLL3 targeting in these populations. However, previous efforts have been limited by lack of stratification by tumor grade, primary site, and/or limited inclusion of G3 tumors. Here, we demonstrate DLL3 expression in 38% of G3 GEP NETs, and an association between DLL3 expression and poor outcomes among G3 PanNETs. In concert, two recent studies noted DLL3 expression in 16%^32^ and 53%^31^ of G3 GEP NETs, respectively, with minimal expression in G1 and G2 tumors.^31,32^ Together, our data identify DLL3-expressing G3 PanNETs as a robust subset of well-differentiated GEP NETs marked by poor outcomes in which DLL3 therapy development should be highly considered.

Through therapeutic efforts across NENs, the degree of DLL3 IHC expression required for actionability of DLL3-targeted therapies has remained uncertain. As above, varied cutoffs for positive^30-32,36^ and high-level^31^ expression have been proposed across NENs. In addition, whereas responses have been observed to tarlatamab in patients with SCLC and no DLL3 IHC expression,^6^ patients with extrapulmonary NECs treated with obrixtamig appear to require >50% tumor cell (DLL3-high) expression for response^37^—notably, 32 of 78 (41%) GEP NECs and 10 of 52 (19%) G3 PanNETs in our data set met this criteria for DLL3-high expression. However, the degree of IHC signal required for activity of DLL3-targeted therapies may vary significantly by agent and across therapeutic classes. Ultimately, DLL3 PET imaging may have better potential to guide treatment selection using real-time functional assessment of DLL3 status across all sites of disease, rather than relying on single-site biopsies, which may not reflect DLL3 expression heterogeneity and interval changes over time. In addition, although limited by sample size, it is noteworthy that the three patients with the highest tumor uptake of the DLL3 PET tracer in our study had variable DLL3 IHC H-scores of 270, 120, and 90, respectively (Table 2), bringing into question the role of IHC from a single tumor tissue sample and suggesting a role for DLL3 PET imaging as an orthogonal and potentially more comprehensive marker for treatment selection.

Patients with G3 PanNETs in our study appeared to exhibit relatively lower DLL3 IHC and PET SUV_max_ values, and higher degrees of intertumoral heterogeneity, compared with patients with GEP NECs. Although this certainly may affect the role of DLL3-targeted therapies in this population, these observations further suggest a role for functional PET imaging as an orthogonal marker for patient selection. In addition, the three cases of G3 PanNETs with [^89^Zr]Zr-DFO-SC16.56 PET tracer uptake in our study displayed variable degrees of concurrent SSTR avidity across tumor lesions (Fig 4 and Data Supplement, Fig S6B). As radioimmunoconjugates targeting DLL3 can be readily converted into therapeutic agents using radionuclides including Lutetium-177^10,11^ or Actinium-225,^10^ our findings that tumor DLL3 expression and SSTR PET avidity may not be mutually exclusive invoke the intriguing possibility of either sequential or combinatorial targeting with DLL3 and SSTR radioligand therapies in PanNETs guided by functional imaging.

Finally, given multiple classes of DLL3-targeted therapies in development, including TCEs,^6-8^ antibody-drug conjugates,^9^ and radioligands,^10,11^ understanding mechanisms of response and acquired resistance to each class of therapies is critical. Correspondingly, whether patients with previous exposure to a DLL3-targeted therapy may benefit from rechallenge with a DLL3-targeted therapy of a different class at progression, and optimal sequencing of these therapies, remains unclear, as resistance could proceed through selection for DLL3-negative subclones, or other tumor-intrinsic or extrinsic mechanisms. In our patient with acquired resistance to a previous DLL3 TCE, high-level functional DLL3 PET avidity was retained at all sites of disease at progression (Figs 3F and 3I), suggesting a role for rechallenge with a different class of DLL3-directed therapy. Further work by each class of agents is needed to explore these concepts, with DLL3 immunoPET-CT imaging potentially of value for patient selection in these scenarios.

Limitations of our study included single-center design with a limited number of [^89^Zr]Zr-DFO-SC16.56 PET scans performed. For IHC analyses, we included data from whole slides and TMAs to increase power. Although a potential limitation related to differences in capturing intratumoral heterogeneity, excellent concordance between slides and TMA blocks has been demonstrated for evaluation of DLL3 IHC positivity in previous similar studies.^31^ In the advanced GEP NEC cohort, three of 46 patients with DLL3 IHC-positive disease received DLL3-targeted therapies in the later-line setting, possibly affecting OS analyses. In addition, in the advanced PanNET cohort, our ability to retrospectively evaluate DLL3 IHC as a prognostic marker was limited by nonuniform treatment strategies across patients, including different sequencing of systemic therapies and nonuniform administration of local therapies, and the assumption that DLL3 status is stable over time in the setting of advanced disease. Recognizing these limitations, however, the association of DLL3 positivity and shorter OS among PanNETs was retained through multiple sensitivity analyses.

In summary, we describe frequent DLL3 IHC expression in high-grade PanNETs and poorly differentiated GEP NECs, nominating these tumor types as key populations for the development of DLL3-targeted therapies. Our results demonstrate the ability of [^89^Zr]Zr-DFO-SC16.56 PET imaging to successfully delineate DLL3-expressing tumors in patients with GEP NENs, underscoring its potential as a targeted diagnostic tool. As further therapeutic efforts progress, functional DLL3 PET imaging may emerge as comprehensive biomarker for patient selection for novel DLL3-targeted therapies, which have the potential to improve outcomes in patients with high-grade GEP NENs.

## Data Availability

A data sharing statement provided by the authors is available with this article at DOI https://doi.org/10.1200/PO-25-00724.
